# *In vivo* activity of Nisin A and Nisin V against *Listeria monocytogenes* in mice

**DOI:** 10.1186/1471-2180-13-23

**Published:** 2013-02-01

**Authors:** Alicia Campion, Pat G Casey, Des Field, Paul D Cotter, Colin Hill, R Paul Ross

**Affiliations:** 1 Department of Microbiology, University College Cork, Cork, Ireland; 2 Teagasc, Moorepark Food Research Centre, Fermoy, Co, Cork, Ireland; 3 Alimentary Pharmabiotic Centre, University College Cork, Cork, Ireland

**Keywords:** Antimicrobial, Lantibiotic, Bacteriocin, Peptide engineering, Mutagenesis, Nisin

## Abstract

**Background:**

Lantibiotics are post-translationally modified antimicrobial peptides, of which nisin A is the most extensively studied example. Bioengineering of nisin A has resulted in the generation of derivatives with increased *in vitro* potency against Gram-positive bacteria. Of these, nisin V (containing a Met21Val change) is noteworthy by virtue of exhibiting enhanced antimicrobial efficacy against a wide range of clinical and food-borne pathogens, including *Listeria monocytogenes.* However, this increased potency has not been tested *in vivo*.

**Results:**

Here we address this issue by assessing the ability of nisin A and nisin V to control a bioluminescent strain of *Listeria monocytogenes* EGDe in a murine infection model.

More specifically, Balb/c mice were infected *via* the intraperitoneal route at a dose of 1 × 10^5^ cfu/animal and subsequently treated intraperitoneally with either nisin V, nisin A or a PBS control. Bioimaging of the mice was carried out on day 3 of the trial. Animals were then sacrificed and levels of infection were quantified in the liver and spleen.

**Conclusion:**

This analysis revealed that nisin V was more effective than Nisin A with respect to controlling infection and therefore merits further investigation with a view to potential chemotherapeutic applications.

## Background

**Figure 1:**
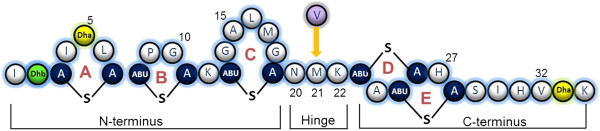
**The structure of nisin A showing the location of the N-terminal domain, containing one lanthionine and two (β-methyl) lanthionine rings (A, B, and C) linked to the C-terminal intertwined rings (D and E) by a flexible hinge region. **Post-translational modifications are highlighted as follows: dehydroalanine (Dha); dehydrobutyrine (Dhb); lanthionine (A-S-A) and (β-methyl) lanthionine (Abu-S-A). Standard residues are represented in the single letter code. Arrow indicates location of the methionine to valine substitution (M21V) in nisin V.

Lantibiotics are ribosomally synthesized peptides produced by Gram-positive bacteria that frequently exhibit potent antimicrobial activities against other bacteria. Nisin A (nisin) is the most intensively investigated lantibiotic, and was first discovered in 1928 [1]. It has a long history of safe use in the food industry and is approved by the US Food and Drug Administration, by WHO and by the EU (as natural food preservative E234) [2-4]. Nisin exhibits antimicrobial activity against many Gram-positive bacteria, including food-borne pathogens such as *Listeria monocytogenes* and *Staphylococcus aureus*. Extensive post-translational modifications are carried out during the biosynthesis of the active 34 amino acid peptide. Specifically, serine and threonine residues in the pro-peptide region are enzymatically dehydrated to dehydroalanine and dehydrobutyrine (Dha and Dhb), respectively. Lanthionine (Lan) and β-methyllanthionine (MeLan) ring structures are generated through the interaction of cysteine with Dha and Dhb, respectively [5-7] (Figure 1). The N-terminal domain, containing one Lan and two meLan rings (A, B, and C) is linked to the C-terminal intertwined rings (D and E) by a flexible hinge region. The antibacterial activity of nisin is exerted *via* a dual action through the activity of the different domains. The N-terminal domain binds to the pyrophosphate moiety of lipid II, inhibiting its transport to the developing cell wall and therefore interfering with cell wall biosynthesis [8]. This binding also facilitates pore formation by the C-terminal domain within the cell membrane, resulting in the loss of solutes from the bacterial cell [9,10]. 

As a result of their highly potent biological activities, lantibiotics have the potential to be employed as novel antimicrobials to combat medically significant bacteria and their multi-drug resistant forms [11-13]. Currently, a number of lantibiotics are under investigation for clinical use. NVB302, a semi-synthetic derivative of actagardine, is in stage I clinical trials with a view to treat infections caused by the hospital-acquired bacteria *Clostridium difficile*[14]. Similarly, microbisporicin (under the commercial name NAI-107), which targets several multi-drug resistant (MDR) bacteria, is in late pre-clinical trials [15]. In models of experimental infection involving mice and rats, the efficacy of microbisporicin *in vivo* was found to be comparable or superior to reference compounds (vancomycin and linezolid) in acute lethal infections induced with several MDR microbes, including methicillin resistant *Staphylococcus aureus* (MRSA), penicillin-intermediate *Streptococcus pneumonia* and vancomycin resistant enterococci (VRE) [16]. Another lantibiotic, mutacin 1140 (produced by *Streptococcus mutans*) is also undergoing pre-clinical trials [17]. Furthermore, a study involving the two peptide lantibiotic, lacticin 3147, has recently demonstrated its ability to prevent systemic spread of *S. aureus* in a murine infection model [18].

Nisin also displays potent *in vitro* activity against multi-drug resistant pathogens such as MRSA, vancomycin-intermediate and -heterogeneous *S. aureus* (VISA and hVISA, respectively) and VRE, [19-21] while natural variants such as nisin F also show potential in this regard [22]. Notably, several studies have also demonstrated the *in vivo* efficacy of nisin A, [23-25] nisin Z, [26,27] and Nisin F [28,29]. Indeed, nisin F was recently shown to successfully treat respiratory disease caused by *S. aureus* K in immunocompromised Wistar rats [28]. These animals were infected intranasally with 4 × 10^5^ *S. aureus* cells prior to treatment with nisin F, also *via* the nasal route. Furthermore, nisin F was found to control the growth of *S. aureus* for up to 15 minutes in mice when injected into the peritoneal cavity [29]. Animals were dosed with 1 × 10^8^ *S. aureus* cells intraperitoneally and subsequently treated with nisin F, also *via* the intraperitoneal route. In a subsequent study, Nisin F-loaded brushite cement was shown to prevent the growth of *S. aureus* Xen 36 [30]. The brushite cement was subcutaneously implanted into mice and infected with 1 × 10^3^ *S. aureus* cells. Release of nisin F from the bone cement prevented *S. aureus* infection for 7 days.

Despite the potency of nisin and its natural variants, the gene encoded nature of these antimicrobials facilitates bioengineering thereof with a view to enhancing potency [31]. Indeed, bioengineering of the hinge region of nisin A has been particularly successful in generating variants with enhanced potency against Gram-positive pathogens [32,33]. One particular derivative, M21V (also known as nisin V), exhibits an *in vitro* activity against *L. monocytogenes* (the causative agent of listeriosis), and indeed other pathogens, which is superior to that of nisin A [34]. While these laboratory-based studies demonstrate the enhanced potency of nisin V against all Gram-positive bacteria tested thus far, it is not known if this enhancement is also evident *in vivo*. In this study, we address this issue by comparing the efficacy of nisin A and nisin V against a *lux*-tagged strain of *L. monocytogenes* (EGDe::pPL2*lux*pHELP) using a murine infection model and, ultimately, demonstrate the greater efficacy of the bioengineered peptide in controlling infection.

## Results/discussion

**Figure 2:**
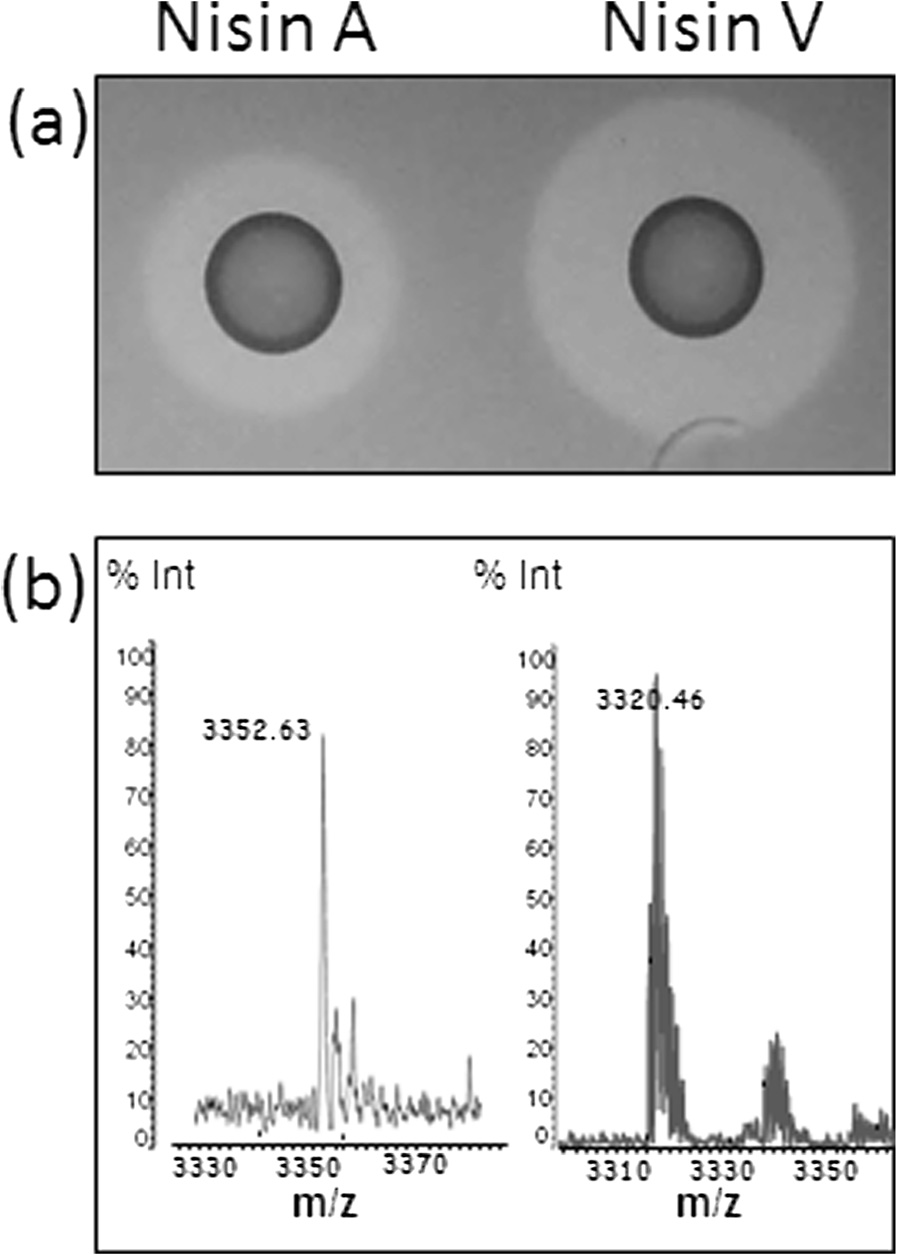
**Deferred antagonism assay and mass spectrometry analysis of nisin A and nisin V. (a)** Inhibition of growth of *L. monocytogenes* EGDe::pPL2*lux*pHELP by the nisin A producing strain *L. lactis* NZ9700 and the nisin V producing strain *L. lactis* NZ9800*nisA*::M21V. **(b)** Mass spectrometry analysis of the nisin A (3353 amu) and nisin V (3321 amu) peptides produced by the bacterial strains *L. lactis* NZ9700 and *L. lactis* NZ9800*nisA*::M21V, respectively.

The ability of nisin A and nisin V to control a *L. monocytogenes* infection in a murine peritonitis model was investigated. Analysis was carried out through bioluminescent imaging of the pathogen in living mice and through the microbiological analysis of organs when mice were sacrificed. Bioluminescence is achieved through the use of a strong constitutive promoter (P_help_ [highly expressed *Listeria* promoter]) driving expression of the *lux* genes of *P. luminescens* integrated into the chromosome of *L. monocytogenes* EGDe [35]. The resulting strain *L. monocytogenes* EGDe::pPL2*lux*pHELP is a strong light-emitter, making it easier to follow *in vivo* using live *in vivo* imaging systems (IVIS). Prior to commencement of the study, the *in vitro* sensitivity of *L. monocytogenes* EGDe::pPL2*lux*pHELP was assessed *via* deferred antagonism assays using nisin A and nisin V producing strains and classical broth-based minimum inhibitory concentration assays (MIC) using purified peptide in each case. Results of deferred antagonism assays with *L. monocytogenes* EGDe::pPL2*lux*pHELP revealed that the nisin V producing strain exhibited increased bioactivity (the combined impact on production and activity) compared to that of *L. lactis* NZ9700 (nisin A producing strain) (Figure 2a). This was in close agreement with previous studies highlighting the similar production levels but increased specific activity of nisin V compared to nisin A [32]. Mass spectrometry analysis of purified nisin A and nisin V peptides confirmed that peptides of correct mass were produced (nisin A - 3353 Da; nisin V- 3321 Da) (Figure 2b). The peptides differ by 32 Da, consistent with the methionine21 to valine (M21V) change of the hinge region of the peptide. Following purification, the specific activity of nisin A and nisin V was tested against *L. monocytogenes* EGDe::pPL2*lux*pHELP using minimum inhibitory concentration (MIC) assays. Nisin A was found to be inhibitory at concentrations of 12.57 mg/L (Table 1), which is consistent with the previously established MIC for the non-*lux* tagged parent strain (*L. monocytogenes* EGDe) [34]. Nisin V was found to be two-fold more active against *L. monocytogenes* EGDe::pPL2*lux*pHELP, with an MIC of 6.22 mg/L. Indeed, the superior activity of nisin V was also confirmed against a number of field and clinical strains of *L. monocytogenes*, where nisin V exhibited at least a two-fold improvement against all nisin A-resistant strains (Table 1). 

**Table 1:** ***In vitro *activity of nisin A and nisin V against *L. monocytogenes *strains as determined by minimum inhibitory concentration assays**^**a**^

**Strain**	**Equivalent name**	**Source/Reference**	**Nisin A mg/L (μM)**	**Nisin V mg/L (μM)**
EGDe::pPL2*lux*pHELP		[35]	12.57 (3.75)	6.22 (1.875)
33028^b^	OB001102	Food	50.28 (15)	24.90 (7.5)
33077^b^	98-18140	Bovine tissue	50.28 (15)	24.90 (7.5)
33225^b^	LMB0455	Unknown	25.14 (7.5)	12.45 (3.75)
F4565^c^	33410, FSLN3-008	Clinical (Los Angeles, California outbreak, 1985)	12.57 (3.75)	6.22 (1.875)
CD1038^d^		Pork sausage	50.28 (15)	12.45 (3.75)

^a^The standard deviation is 0 because of identical triplicate results.

^b^Strain acquired from Todd Ward (Agricultural Research Service, U.S. Department of Agriculture).

^c^Strain acquired from Martin Wiedmann (International Life Sciences Institute).

^d^Strain acquired from Catherine Donnelly (Department of Nutrition and Food Sciences, University of Vermont).

**Figure 3:**
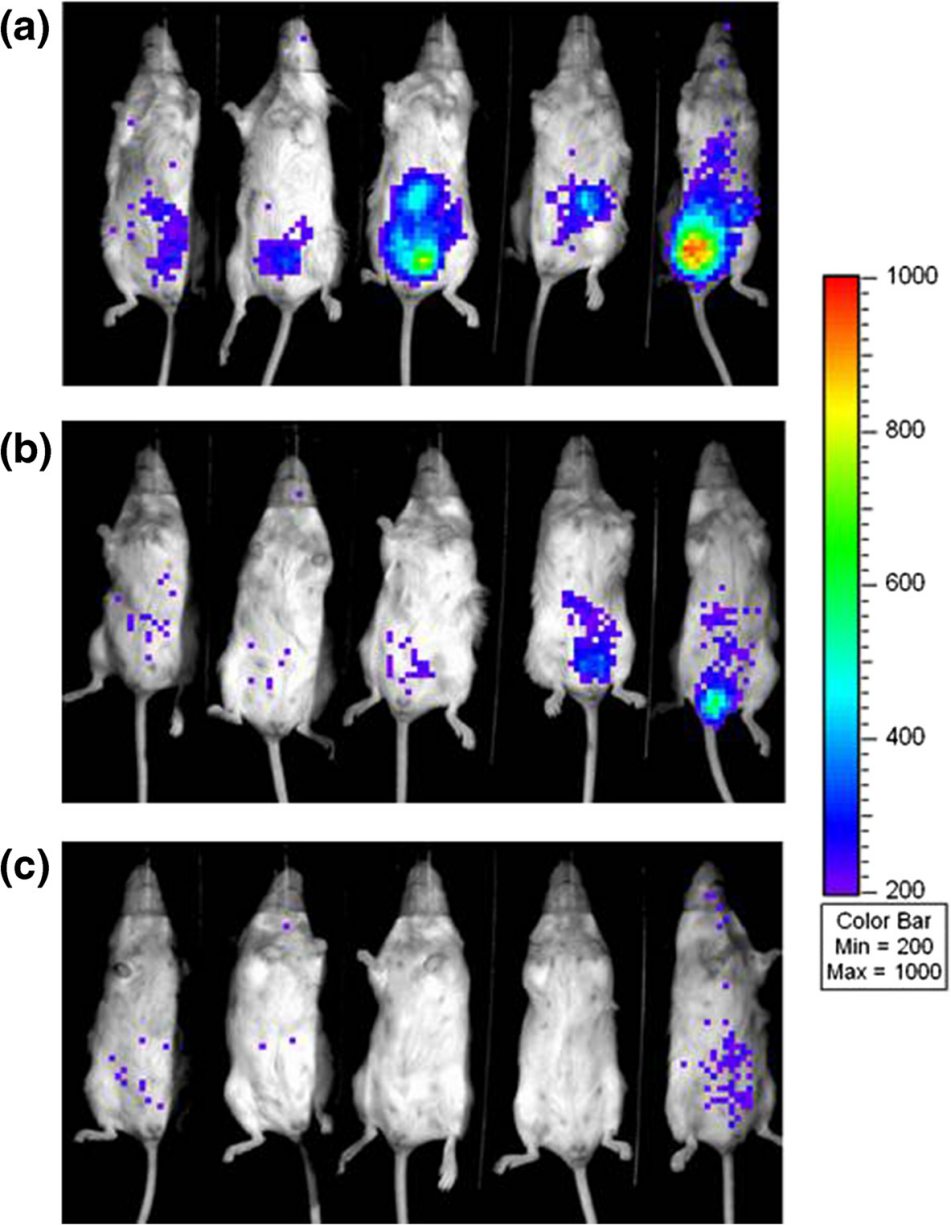
**Analysis of effect of nisin A and nisin V on *Listeria *infection in mice 3 days after intraperitoneal infection with 1 × 10**^**5 **^**CFU *Listeria monocytogenes *EGDe::pPL2*lux*pHELP. **Luminescence observed in animals injected with **(a)** phosphate buffered saline (PBS) **(b)** 58.82 mg/kg nisin A and **(c)** 58.82 mg/kg nisin V 30 minutes after *Listeria* infection.

**Figure 4:**
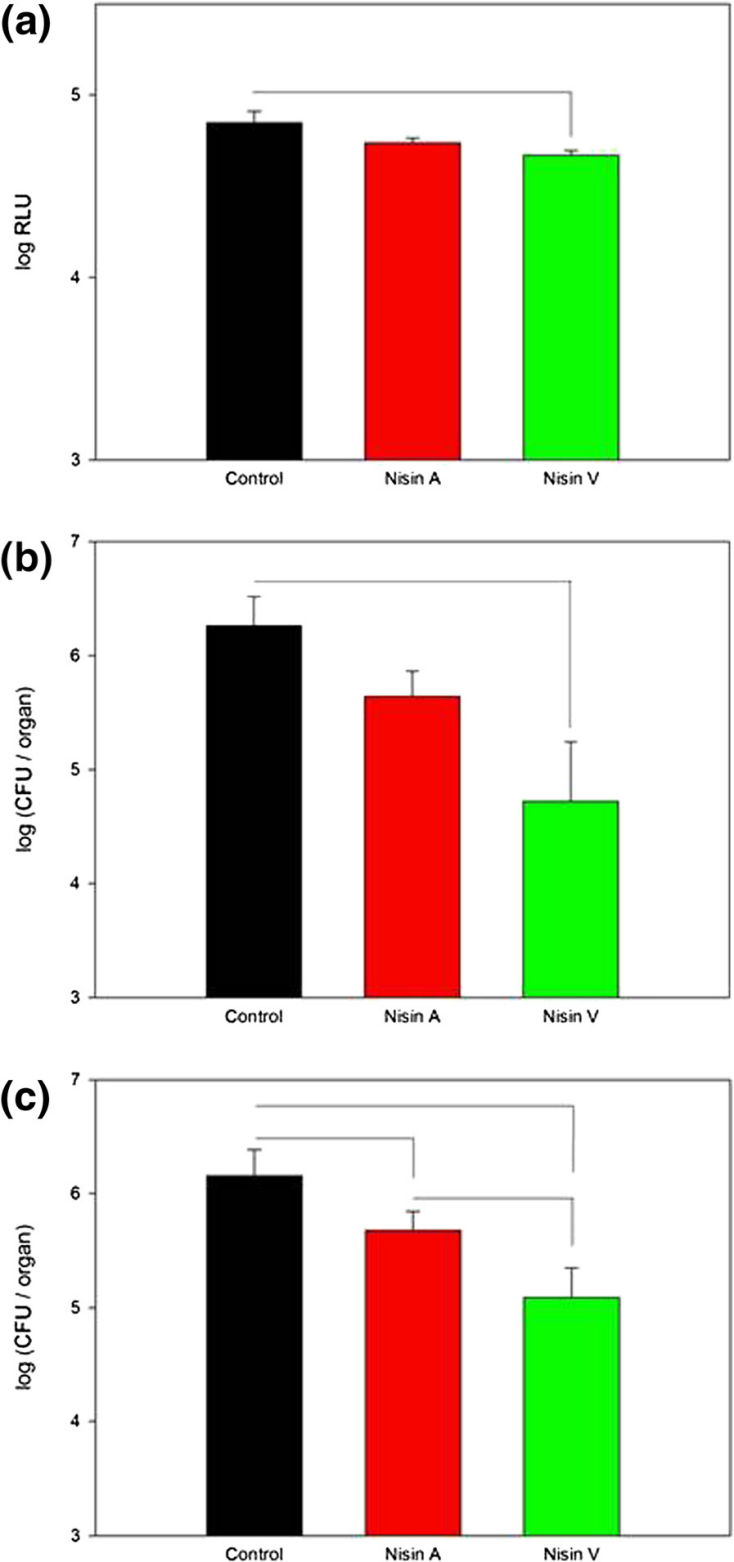
**(a) Relative light unit (RLU) counts in mice 3 days after intraperitoneal infection with 1 × 10**^**5 **^**CFU *L. monocytogenes EGDe::pPL2luxpHELP. *(b)** CFU data from livers and **(c)** spleens of infected mice. Lines connecting groups indicate statistically significant differences between those groups (*P* < 0.05).

For the *in vivo* study, mice were infected *via* the intraperitoneal route with 1 × 10^5^ cfu of *L. monocytogenes* EGDe::pPL2*lux*pHELP and at 30 minutes post infection were treated intraperitoneally with doses of either nisin A (58.82 mg/kg), nisin V (58.82 mg/kg) or PBS (negative control). On day three of the trial, IVIS imaging was used to quantify the level of infection through the detection of light emitted from the pathogen within the mice (Figure 3). While the initial image suggested that nisin A had reduced the amount of luminescence detected (relative light units or RLU), the difference was not statistically significant compared to the PBS-treated control group (Figure 4a). However, a statistically significant reduction (*P* = 0.044) in RLU measurements was observed in the nisin V treated group when compared to the PBS control group (Figure 4a). These results provide the first evidence of the enhanced *in vivo* efficacy of nisin V relative to nisin A. In addition, microbiological analysis of the liver and spleen was determined after the mice were euthanized. While no statistical difference in listerial numbers was observed in the liver between the nisin A and PBS-containing control groups, average pathogen numbers were significantly lower (*P* = 0.018) by over 1 log in the livers of the nisin V-treated groups (4.70 ± 0.5 log cfu) compared to the control group (6.27 ± 0.25 log cfu) (Figure 4b). Analysis of spleens further highlighted the ability of nisin V with respect to controlling *L. monocytogenes* EGDe::pPL2*lux*pHELP infection. In contrast to the liver-related results, spleen cfu counts revealed that nisin A administration had significantly reduced *Listeria* numbers (5.7 ± 0.17 log cfu) (*P* < 0.015) compared to the control group (6.2 ± 0.2 log cfu) (Figure 4c). However, the number of *Listeria* cells in the spleens of nisin V treated animals was significantly lower again, at 5.1 ± 0.25 log cfu, (*P* < 0.015) than that of the other groups (Figure 4c). While the application of lantibiotics in this way to control *Listeria in vivo* is novel, there have been previous successes with linear non-lantibiotic bacteriocins. Indeed, the class IIA bacteriocins, piscicolin 126 and pediocin PA-1 have been shown to effectively control *L. monocytogenes in vivo*[36,37]. 

Although, nisin A displays relatively low cytotoxicity towards intestinal epithelial cells *in vitro*[38] and shows no developmental toxicity in rat models [39], the cytotoxicity of nisin V would have to be investigated further before consideration for use in the clinical setting. However, the fact that nisin V lacks haemolytic activity, even at concentrations of 500 mg/L, and differs from nisin A by just one amino acid may mean that a certain amount of read-across will be permitted and a reduced panel of cytoxicity tests could be sufficient to advance commercial applications. In addition, the success with which bioengineering-based strategies have been employed to enhance its solubility [40], stability [41], diffusion [42] and antimicrobial activity and spectra [32,43,44] would suggest that other derivatives can be generated to further improve upon the functional and pharmokinetic properties of nisin. Alternatively, the use of nisin V in combination with other antimicrobials, such as lysozyme and lactoferrin [28], may also further enhance *in vivo* efficacy.

## Conclusions

This study is the first in which the *in vivo* efficacy of a bioengineered nisin derivative has been assessed. The results revealed that nisin V was more effective than nisin A with respect to controlling infection with *L. monocytogenes* in mice. Significantly, the results validate the use of bioengineering-based strategies for peptide improvement and design and also highlight the potential of nisin V as a chemotherapeutic agent. Enhanced nisins could be especially relevant in situations where traditional antibiotic therapy has failed or where safety issues may predominate. Importantly, the safety of nisin has been well established with, for example, a 90-day oral toxicity study involving rats fed a diet containing nisin A reporting a no-observed-adverse-effect level of approximately 3000 mg/kg/day [45]. Preliminary studies with nisin V revealed a lack of haemolytic activity, even at concentrations of 500 mg/L (D. Field unpublished results).

In conclusion, this study has determined that the enhanced potency of nisin V over nisin A is maintained *in vivo* against the foodborne pathogen *L. monocytogenes* EGDe and suggests that nisin V is a promising candidate as a therapeutic agent.

## Methods

### Bacterial strains and growth conditions

*Lactococcus lactis* NZ9700 and *L. lactis* NZ9800*nisA*::M21V strains were cultured in M17 broth (Oxoid) supplemented with 0.5% glucose (GM17) and GM17 agar at 30°C. Field isolates of *Listeria monocytogenes* and *Listeria monocytogenes* EGDe::pPL2*lux*pHELP, which harbours the *luxABCDE* operon of *P. luminescens* integrated into the chromosome at a single site [35], was grown in Brain Heart Infusion (BHI) broth (Oxoid) or BHI agar at 37°C.

### Nisin purification

Purification of wild type nisin A and the derivative nisin V were carried out as described previously [34]. Briefly, overnight cultures of the wild type nisin A producing strain *L. lactis* NZ9700 [46] and the nisin V producing variant *L. lactis* NZ9800*nisA*::M21V [34] were grown in GM17 broth at 30°C and were subsequently inoculated into two litres of purified TY broth at 1% and incubated overnight at 30°C. The culture was centrifuged at 7,000 r.p.m. for 20 minutes and the supernatant retained. The supernatant was applied to a 60 g Amberlite bead (Sigma) column, which was subsequently washed with 500 ml of 30% ethanol and the inhibitory activity eluted in 500 ml of 70% isopropanol 0.1% trifluoroacetic acid (TFA). The cell pellet was resuspended in 300 ml of 70% isopropanol 0.1% TFA and magnetically stirred for 3 hours at room temperature. The cells were removed by centrifugation at 7,000 r.p.m. for 20 minutes and the supernatant retained. The isopropanol was evaporated off using a rotary evaporator (Buchi) to a volume of 160 ml and the sample pH adjusted to approximately 4.2. The sample was applied to a 10 g (60 ml) Varian C-18 Bond Elut Column previously pre-equilibrated with HPLC water and methanol. The column was washed with 120 ml of 30% ethanol and the inhibitory activity eluted in 60 ml of 70% isopropanol 0.1% TFA. Six millilitres of the lantibiotic preparation was concentrated to 1 ml through the removal of the isopropanol by rotary evaporation and applied to a Phenomenex C12 reverse-phase (RP)-HPLC column, previously equilibrated with 25% isopropanol 0.1% TFA. The column was then developed in a gradient of 30% isopropanol 0.1% TFA to 60% isopropanol 0.1% TFA from 10 to 45 minutes at a flow rate of 2.1 ml/min. Fractions containing nisin A and nisin V peptides were collected and subjected to Mass Spectrometry with a Shimadzu Biotech MALDI-TOF Mass Spectrometer (AXIMA-CFR plus model).

### Bioassays for antimicrobial activity

Deferred antagonism assays were carried out as previously described [34]. Briefly, 5 μl of fresh overnight cultures of *L. lactis* NZ9700 and *L. lactis* NZ9800*nisA*::M21V were spotted and allowed to grow on GM17 agar overnight. The colonies were subjected to 30 mins UV radiation prior to overlaying with BHI agar (0.75% w/v agar) seeded with the indicator strain *L. monocytogenes* EGDe::pPL2*lux*pHELP. The plates were then incubated at 37°C overnight and relative zone size compared.

### Minimum inhibitory concentration (MIC) assays

The MIC of nisin A and nisin V against *Listeria monocytogenes* EGDe::pPL2*lux*pHELP and several field isolates of *Listeria monocytogenes* was carried out in triplicate as previously described [34]. Briefly, prior to the addition of purified peptides, the 96-well microtitre plates were pre-treated with 200 μl of phosphate buffered saline (PBS) containing 1% (w/v) bovine serum albumin (BSA) and incubated at 37°C for 30 min. Wells were washed with PBS and left to dry before the addition of 100 μl BHI broth. *L. monocytogenes* strains were grown overnight in BHI broth at 37°C, subcultured into fresh BHI broth and grown to log phase (OD_600nm_ of 0.5). The cultures were diluted to a concentration of 1 × 10^5^ cfu/ml in a 0.2 ml volume. The purified peptides were resuspended in BHI broth to a stock concentration of 60 μM and adjusted to a 15 μM starting concentration. Two-fold dilutions of the peptides were made in the 96-well plates, which were subsequently inoculated with the bacterial strains and incubated at 37°C for 16 hours. The minimum inhibitory concentration (MIC) was read as the minimum peptide concentration inhibiting visible growth of the bacterial strains.

### Inoculum preparation

*L. monocytogenes* EGDe was grown overnight in BHI broth at 37°C from an isolated colony growing on a BHI agar plate containing 7.5 mg/L chloramphenicol. The overnight culture was diluted in order to facilitate its administration in a dose of 1 × 10^5^ cfu/200 μl PBS.

### Mouse model

All procedures involving animals were approved by the UCC Animal Experimentation Ethics Committee and carried out by a licensed individual with an ethical approval number of 2011/017. For the *L. monocytogenes* murine model, 15 Balb/c female mice (7 weeks old, 15 g ± 2 g in weight) were divided into three groups (A, B and C) with each group containing 5 mice. At T_0_ on day 1, all groups were infected with 1 × 10^5^ viable cells of *L. monocytogenes* EDGe::pPL2*lux*pHELP in a 200 μl dose of PBS *via* the intraperitoneal (I.P.) route. At T_0.5hrs_, mice in group A were administered PBS (control), group B were treated with nisin A (58.82 mg/kg) and group C treated with nisin V (58.82 mg/kg). Both PBS and the nisin peptides were administered in 200 μl doses *via* the I.P. route. On day 3, the mice were anaesthetised using a mixture of aerosolised isoflurane and oxygen. Bioluminescence was monitored using an IVIS® Imaging System 100 series (Xenogen Corporation, Almeda, CA) with a 5 minute exposure time. Immediately afterward, the mice were euthanized and the livers and spleens were extracted. The organs were mechanically disrupted and serial dilutions made which were subsequently plated in 100 μl volumes on BHI agar plates containing chloramphenicol 7.5 mg/L in order to enumerate *L. monocytogenes* present in each organ.

### Luminescence quantification

IVIS imaging software was used to carry out quantification of luminescence. Bioluminescence emitted from the infection site was measured as total counts across the region of interest (designated relative light units – “RLU”) and was averaged across all groups of mice. The reduction in luminescence was quantified and represents a comparison with the luminescence from mice administered PBS control at the same time point.

### Statistical analysis

CFU and RLU data was transformed to log_10_ prior to analysis. All comparisons were based on the mean ± standard error of the mean (SEM). Parametric data was analysed using one way analysis of variance (ANOVA) with post hoc comparison using the Student-Newman-Keuls method. Non-parametric data was analysed by the Kruskal–Wallis one way ANOVA with post hoc comparison as above. *P* < 0.05 was considered to be significant in all cases.

## Competing interests

The authors declare that they have no competing interests.

## Authors’ contributions

AC designed experiments, carried out nisin purification, antimicrobial activity bioassays, MIC assays and inoculum preparation and drafted the manuscript. PGC conducted and provided mouse model analysis. DF contributed to the conduct of experiments and reviewing the manuscript. PDC, CH and RPR conceived the study and participated in its design and implementation and reviewed the manuscript. All authors read and approved the final manuscript.

## References

[B1] Rogers LA Whittier EO Limiting factors in the lactic fermentation J Bacteriol 1928 16 211 229 16559334 10.1128/jb.16.4.211-229.1928 PMC375023

[B2] Chen H Hoover DG Bacteriocins and their food applications Comprehensive Rev Food Sci Food Safety 2003 2 82 100 10.1111/j.1541-4337.2003.tb00016.x 33451234

[B3] Delves-Broughton J Nisin and its uses as a food preservative Food Technol 1990 44 100 117

[B4] Guinane CM Cotter PD Hill C Ross RP Microbial solutions to microbial problems; lactococcal bacteriocins for the control of undesirable biota in food J Appl Microbiol 2005 98 1316 1325 10.1111/j.1365-2672.2005.02552.x 15916645

[B5] de Vos WM Kuipers OP van der Meer JR Siezen RJ Maturation pathway of nisin and other lantibiotics: post-translationally modified antimicrobial peptides exported by Gram-positive bacteria Mol Microbiol 1995 17 427 437 10.1111/j.1365-2958.1995.mmi_17030427.x 8559062

[B6] Sahl H Jack R Bierbaum G Biosynthesis and biological activities of lantibiotics with unique post-translational modifications Eur J Biochem 1995 230 827 853 10.1111/j.1432-1033.1995.tb20627.x 7601145

[B7] Bierbaum G Sahl HG Lantibiotics: mode of action, biosynthesis and bioengineering Curr Pharm Biotechnol 2009 10 2 18 10.2174/138920109787048616 19149587

[B8] Hsu ST Breukink E Tischenko E Lutters MA de Kruijff B Kaptein R Bonvin AM van Nuland NA The nisin-lipid II complex reveals a pyrophosphate cage that provides a blueprint for novel antibiotics Nat Struct Mol Biol 2004 11 963 967 10.1038/nsmb830 15361862

[B9] Wiedemann I Breukink E van Kraaij C Kuipers OP Bierbaum G de Kruijff B Sahl HG Specific binding of nisin to the peptidoglycan precursor lipid II combines pore formation and inhibition of cell wall biosynthesis for potent antibiotic activity J Biol Chem 2001 276 1772 1779 11038353 10.1074/jbc.M006770200

[B10] Wiedemann I Benz R Sahl HG Lipid II-mediated pore formation by the peptide antibiotic nisin: a black lipid membrane study J Bacteriol 2004 186 3259 3261 10.1128/JB.186.10.3259-3261.2004 15126490 PMC400633

[B11] Cotter PD Hill C Ross RP Bacterial lantibiotics: strategies to improve therapeutic potential Curr Protein Pept Sci 2005 6 61 75 10.2174/1389203053027584 15638769

[B12] Piper C Cotter PD Ross RP Hill C Discovery of medically significant lantibiotics Curr Drug Discov Technol 2009 6 1 18 10.2174/157016309787581075 19275538

[B13] Lawton EM Ross RP Hill C Cotter PD Two-peptide lantibiotics: a medical perspective Mini Rev Med Chem 2007 7 1236 1247 10.2174/138955707782795638 18220976

[B14] New antibiotic compound enters phase I clinical trial http://www.wellcome.ac.uk/News/2011/News/WTVM053339.htm

[B15] Foulston LC Bibb MJ Microbisporicin gene cluster reveals unusual features of lantibiotic biosynthesis in actinomycetes Proc Natl Acad Sci U S A 2010 107 13461 13466 10.1073/pnas.1008285107 20628010 PMC2922176

[B16] Jabes D Brunati C Candiani G Riva S Romano G Donadio S Efficacy of the new lantibiotic NAI-107 in experimental infections induced by MDR Gram-positive pathogens Antimicrob Agents Chemother 2011 55 1671 1676 10.1128/AAC.01288-10 21220527 PMC3067139

[B17] Smith L Hillman J Therapeutic potential of type A (I) lantibiotics, a group of cationic peptide antibiotics Curr Opin Microbiol 2008 11 401 408 10.1016/j.mib.2008.09.008 18848642 PMC2603291

[B18] Piper C Casey PG Hill C Cotter PD The lantibiotic lacticin 3147 prevents systemic spread of *Staphylococcus aureus* in a murine infection model Int J Microbiol 2012 **2012.** 10.1155/2012/806230 PMC3265090 22291709

[B19] Severina E Severin A Tomasz A Antibacterial efficacy of nisin against multidrug-resistant Gram-positive pathogens J Antimicrob Chemother 1998 41 341 347 10.1093/jac/41.3.341 9578160

[B20] Brumfitt W Salton MR Hamilton-Miller JM Nisin, alone and combined with peptidoglycan-modulating antibiotics: activity against methicillin-resistant *Staphylococcus aureus* and vancomycin-resistant enterococci J Antimicrob Chemother 2002 50 731 734 10.1093/jac/dkf190 12407132

[B21] Piper C Draper LA Cotter PD Ross RP Hill C A comparison of the activities of lacticin 3147 and nisin against drug-resistant *Staphylococcus aureus* and Enterococcus species J Antimicrob Chemother 2009 63 546 551 19561147 10.1093/jac/dkp221

[B22] Piper C Hill C Cotter PD Ross RP Bioengineering of a nisin A-producing *Lactococcus lactis* to create isogenic strains producing the natural variants nisin F, Q and Z Microb Biotechnol 2011 4 375 382 10.1111/j.1751-7915.2010.00207.x 21375711 PMC3818996

[B23] Coughlin R Tikofsky L Schulte H Bennett G Rejman J Fisher D Crabb J Schukken Y Lactation mastitistherapy with the nisin-based product MastOut: results of a 125-cow study National Mastitis Council Annual Meeting 2004 43 296 297

[B24] Goldstein BP Wei J Greenberg K Novick R Activity of nisin against *Streptococcus pneumoniae*, *in vitro*, and in a mouse infection model. J Antimicrob Chemother 1998 42 277 278 10.1093/jac/42.2.277 9738856

[B25] Taylor J Hirsch AR Mattick AT The treatment of bovine streptococcal and staphylococcal mastitis with nisin Vet Res 1949 61 197 198

[B26] Cao LT Wu JQ Xie F Hu SH Mo Y Efficacy of nisin in treatment of clinical mastitis in lactating dairy cows J Dairy Sci 2007 90 3980 3985 10.3168/jds.2007-0153 17639009

[B27] Wu J Hu S Cao L Therapeutic effect of nisin Z on subclinical mastitis in lactating cows Antimicrob Agents Chemother 2007 51 3131 3135 10.1128/AAC.00629-07 17606675 PMC2043217

[B28] De Kwaadsteniet M Doeschate KT Dicks LM Nisin F in the treatment of respiratory tract infections caused by *Staphylococcus aureus* Lett Appl Microbiol 2009 48 65 70 10.1111/j.1472-765X.2008.02488.x 19018962

[B29] Brand AM De Kwaadsteniet M Dicks LMT The ability of nisin F to control *Staphylococcus aureus* infection in the peritoneal cavity, as studied in mice Lett Appl Microbiol 2010 51 645 649 10.1111/j.1472-765X.2010.02948.x 21029139

[B30] van Staden AD Brand AM Dicks LMT Nisin F-loaded brushite bone cement prevented the growth of *Staphylococcus aureus in vivo* J Appl Microbiol 2012 112 831 840 10.1111/j.1365-2672.2012.05241.x 22268790

[B31] Field D Hill C Cotter PD Ross RP The dawning of a ‘Golden era’ in lantibiotic bioengineering Mol Microbiol 2010 78 1077 1087 10.1111/j.1365-2958.2010.07406.x 21091497

[B32] Field D O’Connor PM Cotter PD Hill C Ross RP The generation of nisin variants with enhanced activity against specific Gram-positive pathogens Mol Microbiol 2008 69 218 230 10.1111/j.1365-2958.2008.06279.x 18485077

[B33] Carroll J Field D O’ Connor PM Cotter PD Coffey A Hill C Ross RP O’ Mahony J The gene encoded antimicrobial peptides, a template for the design of novel anti-mycobacterial drugs Bioengineered Bugs 2010 1 408 412 10.4161/bbug.1.6.13642 21468208 PMC3056091

[B34] Field D Quigley L O’Connor PM Rea MC Daly K Cotter PD Hill C Ross RP Studies with bioengineered nisin peptides highlight the broad-spectrum potency of nisin V Microb Biotechnol 2010 3 473 486 10.1111/j.1751-7915.2010.00184.x 21255345 PMC3815813

[B35] Riedel CU Monk IR Casey PG Morrissey D O’Sullivan GC Tangney M Hill C Gahan CGM Improved luciferase tagging system for *Listeria monocytogenes* allows real-time monitoring *in vivo* and *in vitro* Appl Environ Microbiol 2007 73 3091 3094 10.1128/AEM.02940-06 17351089 PMC1892880

[B36] Ingham A Ford M Moore RJ Tizard M The bacteriocin piscicolin 126 retains antilisterial activity *in vivo* J Antimicrob Chemother 2003 51 1365 1371 10.1093/jac/dkg229 12716771

[B37] Dabour N Zihler A Kheadr E Lacroix C Fliss I *In vivo* study on the effectiveness of pediocin PA-1 and *Pediococcus acidilactici* UL5 at inhibiting *Listeria monocytogenes* Int J Food Microbiol 2009 133 225 233 10.1016/j.ijfoodmicro.2009.05.005 19541383

[B38] Maher S McClean S Investigation of the cytotoxicity of eukaryotic and prokaryotic antimicrobial peptides in intestinal epithelial cells *in vitro* Biochem Pharmacol 2006 71 1289 1298 10.1016/j.bcp.2006.01.012 16530733

[B39] Gupta SM Aranha CC Reddy KV Evaluation of developmental toxicity of microbicide nisin in rats Food Chem Toxicol 2008 46 598 603 10.1016/j.fct.2007.09.006 17949878

[B40] Liu W Hansen JN Some chemical and physical properties of nisin, a small-protein antibiotic produced by *Lactococcus lactis* Appl Environ Microbiol 1990 56 2551 2558 2119570 10.1128/aem.56.8.2551-2558.1990 PMC184764

[B41] Rollema HS Kuipers OP Both P de Vos WM Siezen RJ Improvement of solubility and stability of the antimicrobial peptide nisin by protein engineering Appl Environ Microbiol 1995 61 2873 2878 7487019 10.1128/aem.61.8.2873-2878.1995 PMC167563

[B42] Rouse S Field D Daly KM O’Connor PM Cotter PD Hill C Ross RP Bioengineered nisin derivatives with enhanced activity in complex matrices Microb Biotechnol 2012 5 501 508 10.1111/j.1751-7915.2011.00324.x 22260415 PMC3815327

[B43] Yuan J Zhang ZZ Chen XZ Yang W Huan LD Site-directed mutagenesis of the hinge region of nisin Z and properties of nisin Z mutants Appl Microbiol Biotechnol 2004 64 806 815 10.1007/s00253-004-1599-1 15048591

[B44] Field D Begley M O’Connor PM Daly KM Hugenholtz F Cotter PD Hill C Ross RP Bioengineered nisin A derivatives with enhanced activity against both Gram positive and Gram negative pathogens PLoS One 2012 7 e46884 10.1371/journal.pone.0046884 23056510 PMC3466204

[B45] Hagiwara A Imai N Nakashima H Toda Y Kawabe M Furukawa F Delves-Broughton J Yasuhara K Hayashi S-M A 90-day oral toxicity study of nisin A, an anti-microbial peptide derived from *Lactococcus lactis* subsp. *lactis*, in F344 rats Food Chem Toxicol 2010 48 2421 2428 10.1016/j.fct.2010.06.002 20621644

[B46] Kuipers OP Beerthuyzen MM Siezen RJ De Vos WM Characterization of the nisin gene cluster *nisABTCIPR* of *Lactococcus lactis*. Requirement of expression of the *nisA* and *nisI* genes for development of immunity Eur J Biochem 1993 216 281 291 10.1111/j.1432-1033.1993.tb18143.x 7689965

